# Urine Bikunin as a Marker of Renal Impairment in Fabry's Disease

**DOI:** 10.1155/2013/205948

**Published:** 2013-06-12

**Authors:** Antonio Junior Lepedda, Laura Fancellu, Elisabetta Zinellu, Pierina De Muro, Gabriele Nieddu, Giovanni Andrea Deiana, Piera Canu, Daniela Concolino, Simona Sestito, Marilena Formato, Gianpietro Sechi

**Affiliations:** ^1^Dipartimento di Scienze Biomediche, University of Sassari, Via Muroni 25, 07100 Sassari, Italy; ^2^Dipartimento di Medicina Clinica e Sperimentale, University of Sassari, Viale San Pietro 10, 07100 Sassari, Italy; ^3^Unità Operativa di Pediatria Universitaria, Azienda Ospedaliera “Pugliese-Ciaccio”, Viale Pio X, 88100 Catanzaro, Italy

## Abstract

Fabry's disease is a rare lysosomal storage disorder caused by the deficiency of **α**-galactosidase A that leads to the accumulation of neutral glycosphingolipids in many organs including kidney, heart, and brain. Since end-stage renal disease represents a major complication of this pathology, the aim of the present work was to evaluate if urinary proteoglycan/glycosaminoglycan excretion could represent a useful marker for monitoring kidney function in these patients at high risk. Quali-quantitative and structural analyses were conducted on plasma and urine from 24 Fabry's patients and 43 control subjects. Patients were sorted for presence and degree of renal impairment (proteinuria/renal damage). Results showed that levels of urine bikunin, also known as urinary trypsin inhibitor (UTI), are significantly higher in patients with renal impairment than in controls. In this respect, no differences were evidenced in plasma chondroitin sulfate isomers level/structure indicating a likely direct kidney involvement. Noteworthy, urine bikunin levels are higher in patients since early symptoms of renal impairment occur (proteinuria). Overall, our findings suggest that urine bikunin level, as well as proteinuria, could represent a useful parameter for monitoring renal function in those patients that do not present any symptoms of renal insufficiency.

## 1. Introduction

Fabry's disease (FD) is a panethnic, X-linked lysosomal storage disorder due to deficiency of *α*-galactosidase A [1]. This lysosomal enzyme normally breaks down neutral glycosphingolipids, particularly globotriaosylceramide (Gb3), catalyzing the hydrolytic cleavage of the terminal molecule of galactose. The consequent accumulation of these glycosphingolipids in many cell types and tissues results in several clinical signs and symptoms [1]. The prevalence of Fabry's disease has been estimated to range from 1 in 117,000 to up to 1 in 40,000, but it might be much higher since it is likely that many patients are not identified, because of either the nonspecificity of clinical features or the scarce suspicion of the clinician for the disease [1]. The *α*-galactosidase A gene (GLA-gene) is located on the long arm of chromosome X in position Xq22, and it has recently been sequenced [1]. More than 400 mutations have been identified so far. Depending on the type of mutation there may be different clinical forms of the disease. In particular, GLA-gene mutations resulting in a total absence of *α*-galactosidase A activity usually lead to a more severe form of FD [1]. Disease manifestations usually start in childhood, with intermittent acroparesthesias, sometimes associated with episodic fever, hypohidrosis, gastrointestinal symptoms, typical vascular skin lesions named angiokeratomas, and corneal opacities [2]. After the 3rd decade of life the progression of the pathology frequently leads to renal damage [3], cardiac manifestations, and high propensity to develop brain ischemic stroke, resulting in decreased life expectancy [4]. In particular, end-stage renal disease, with proteinuria and progressive renal failure, is a major cause of morbidity and mortality in FD. Renal damage seems mainly to be caused by diffuse deposition of glycosphingolipids in glomeruli, tubular system, and vasculature. In routine clinical practice, general proteinuria and microalbuminuria are considered the best biomarkers of FD nephropathy [3], although, recently, many new markers, including Gb3 or specific proteins such as N-acetyl-*β*-D-glucosaminidase and cystatin C, have been suggested to improve decision making [5]. However, the efficacy of all of the biomarkers currently in use for Fabry's nephropathy remains uncertain. 

Several studies evidenced variations in plasma/urine glycosaminoglycans in physiological and pathological conditions. Glycosaminoglycans (GAGs) are linear and complex polysaccharides, composed of a variable number of repeating disaccharide units, each containing a hexuronic acid glycosidically linked to a hexosamine residue. GAGs have been found in many tissues and in biological fluids such as blood, plasma and urine [6]. Plasma GAGs represent components of intact proteoglycans (PGs) mainly of hepatic and endothelial origin, secreted into blood as well as products of tissue PG degradation. Chondroitin sulfate (CS), the main GAG type in plasma, is derived from both the cell surfaces and the extracellular matrix. A portion of CS is covalently bound to a protein core to form bikunin that is principally synthesized and secreted by the liver [7]. CS chains are short, consisting of 12–18 disaccharides units, and present a charge density of about 30–40% as previously reported [8, 9]. Most of circulating bikunin is present as the light chain of Inter-*α*-Inhibitor (I*α*I) family molecules [10]. Bikunin is a serine protease inhibitor and it occurs in plasma as well as in many tissues [10]. It is also excreted in urine and referred to as urinary trypsin inhibitor (UTI). In urine, GAGs consist mainly of heparan sulfate (HS), CS, and, in negligible quantity, dermatan sulfate [6]. In the general population, bikunin has been reported to occur at higher levels in various pathological conditions exhibiting chronic inflammation, including cancer [11], chronic glomerulonephritis [12, 13], kidney transplantation [14], type 1 diabetes [15], and systemic lupus erythematosus [16]. Moreover, we reported variations of urine bikunin levels during the physiological menstrual cycle in fertile women [17]. It has also been suggested that bikunin may be a useful marker for renal damage [18], liver disease [19], and brain contusion [20], suggesting a potential application in patients with FD. Since kidney damage is a frequent and severe complication of FD, we thought of evaluating plasma and urine GAGs levels in this pathology to assess if their urinary excretion could represent a useful early marker of kidney impairment in patients with Fabry's disease. 

## 2. Methods

### 2.1. Samples

Analyses were conducted on fasting blood-plasma and first-morning urine samples from 24 Fabry's patients of both sexes, aged from 20 to 61 years, and 43 age- and gender-matched healthy controls. All FD patients were diagnosed by identifying the mutation in the GLA-gene and showed reduced activity of *α*-galactosidase A enzyme in plasma (Table 1). Among patients, 13 did not present evidence of chronic renal damage (NRD); the other 11, instead, presented, for more than 3 months, pathological abnormalities indicative of chronic renal damage (RD), including abnormalities in blood or urine tests (i.e., serum creatinine; proteinuria/microalbuminuria; and abnormalities in urine sediment) or imaging studies (e.g., renal ultrasound). More in detail, 5 RD patients presented with only proteinuria, whereas 6 presented with overt renal damage. In both FD patients and controls the serum levels of three classic markers of inflammation such as erythrocyte sedimentation rate (ESR) (by means of stopped flow capillary microspectrophotometry), C-reactive protein (CRP) (by means of chemiluminescence assay), and *α*1- and *α*2-globulins (by means of capillary electrophoresis) were evaluated. Informed consent was obtained before enrolment. Institutional review board approval was obtained. The study was conducted in accordance with the ethical principles of the current Declaration of Helsinki.

### 2.2. Plasma CS Isomers Analysis

GAGs isolation was performed by a microanalytic preparative method, as previously described [8]. Briefly, 500 *μ*L of plasma samples was subjected to proteolytic treatment with papain. Plasma CS isomers were isolated by anion exchange chromatography (DEAE-Sephacel) and precipitated with 5 volumes of ethanol at −20°C for 24 h. Subsequently, they were subjected to depolymerization by using chondroitin ABC lyase (0.1 U per 100 *μ*g hexuronic acid), and the unsaturated disaccharides were derivatized with 12.5 mmol/L 2-aminoacridone (AMAC).

Separation of CS-derived unsaturated disaccharides (ΔDi) was performed by fluorophore-assisted carbohydrate electrophoresis (FACE). Images were acquired by means of Gel Doc XR system (Bio-Rad) and analyzed by using Quantity One v4.6.3 software (Bio-Rad). A calibration curve was set up by using home-made CS isomers obtained from a pool of plasma samples, assayed for hexuronate content [21], and subjected to disaccharides analysis [8]. CS levels were reported as *μ*g of hexuronic acid per mL of plasma (*μ*g_UA_/mL), and CS charge density was evaluated as ratio between 4-sulfated Δ-disaccharides (ΔDi-4S) and total unsaturated disaccharides (ΔDi-4S + ΔDi-nonS).

### 2.3. Urine GAGs/Bikunin Analysis

First-morning urine samples (about 50 mL) were collected and, after centrifugation at 3000 g for 15 min at 4°C, the sediment of broken cells or tissues and other solid materials was discarded. Urine GAGs/bikunin containing fraction was obtained by anion exchange chromatography (DEAE-Sephacel resin) as previously described [15]. Briefly, clarified urines were applied to a column packed with about 6 mL of resin, previously equilibrated with a buffer containing 0.02 M Tris-HCl, 0.15 M NaCl (pH 8.6). After exhaustive washing, urinary GAGs/bikunin were eluted with a buffer containing 0.02 M Tris-HCl, 2 M LiCl (pH 8.6), and assayed for hexuronate content by the method of Bitter and Muir, using glucuronic acid as a standard [21]. Hexuronate levels were normalized for the urinary creatinine concentration, formerly determined by the Jaffè method (Sentinel CH, Milan, Italy). Urinary GAGs/bikunin composition was determined by discontinuous electrophoresis on cellulose acetate plates [12–17], according to Cappelletti et al. [22]. Analytes were resolved by three electrophoretic steps in 0.25 M barium acetate running buffer, pH 5.0. Titan III-H cellulose acetate plate (6.0 × 7.5 cm, Helena BioSciences) was first soaked in distilled H_2_O for about 1.5 cm and immediately blotted between filter papers. Then, the opposite end was soaked in 0.1 M barium acetate buffer, pH 5.0, for 5.5 cm, leaving a narrow band (2–4 mm large), apparently dry, where 5 *μ*g as uronic acid of each sample was loaded. Electrophoresis was carried out at 5 mA for about 6 minutes followed by incubation of the plate in 0.1 M barium acetate, pH 5.0, for 2 minutes. The second electrophoretic step was carried out at 15 mA for 14 minutes. Subsequently, the plate was soaked in 0.1 M barium acetate buffer, pH 5.0, containing 15% (v/v) ethanol for 2 minutes. A third electrophoretic step was carried out at 12 mA for 17 minutes. Finally, electrophoretic profiles were detected following 0.1% (w/v) Alcian Blue staining. Images were acquired by means of GS-800 calibrated densitometer (Bio-Rad) and analyzed by using Quantity One v4.6.3 software (Bio-Rad). GAGs were expressed as relative percentages. 

The GAGs/bikunin fractions were assessed for both urine bikunin protein content and presence of urine bikunin fragments by performing SDS-PAGE followed by highly sensitive Coomassie Brilliant Blue G-250 staining (limit of detection: 20 ng of protein) [23] on untreated samples and after chondroitin ABC lyase digestion. The latter was performed in a buffer containing 0.1 M ammonium acetate (pH 8.0) using 0.1 U of chondroitin ABC lyase (Sigma Aldrich) per 100 *μ*g of hexuronate at 37°C overnight. Samples were added with 4X SDS-buffer containing 250 mM Tris (pH 6.8), 8% SDS (w/v), 8% DTT (w/v), 40% glycerol (v/v), and 0.0008% bromophenol blue (w/v) and boiled for 5 minutes before electrophoresis. Urine bikunin was resolved by Tris-glycine SDS-PAGE in 1 mm thick 15% T, 3% C running gel, using a MiniProtean II cell vertical slab gel electrophoresis apparatus (Bio-Rad). Electrophoresis was carried out at 50 V for 15 minutes and subsequently at 150 V until the bromophenol dye front reached the lower limit of the gel. Then, gels were fixed in 30% ethanol (v/v), 2% phosphoric acid (v/v) for 1 h, washed twice in 2% phosphoric acid (v/v) for 10 minutes, equilibrated in 18% ethanol (v/v), 2% phosphoric acid (v/v), and 15% ammonium sulfate (w/v) for 30 minutes, and stained in the same solution containing 0.02% Coomassie Brilliant Blue G-250 (w/v) for 48 h. Gel images were acquired by using GS-800 calibrated densitometer (Bio-Rad) at 63 *μ*m resolution.

### 2.4. Statistical Analysis

Student's *t*-test for unpaired samples was used to compare plasma and urinary GAGs levels between Fabry's patients and control subjects, using the software package Sigma Stat 3 (Systat Software). Pearson's correlation analysis was performed to evaluate the association between plasma CS and UTI levels and between UTI and serum creatinine levels. Significance was set at *P* < 0.05.

## 3. Results

**Figure 1:**
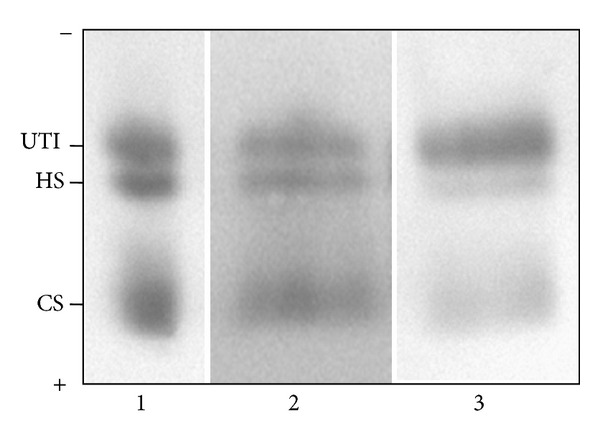
Representative cellulose acetate electrophoretic profiles of urine glycosaminoglycans/UTI from control subjects (lane 2) and Fabry's patients with renal disease (lane 3). Lane 1: mixture of standard GAGs/UTI (UTI: urinary trypsin inhibitor/urine bikunin; HS: heparan sulfate; CS: chondroitin sulfate).

**Figure 2:**
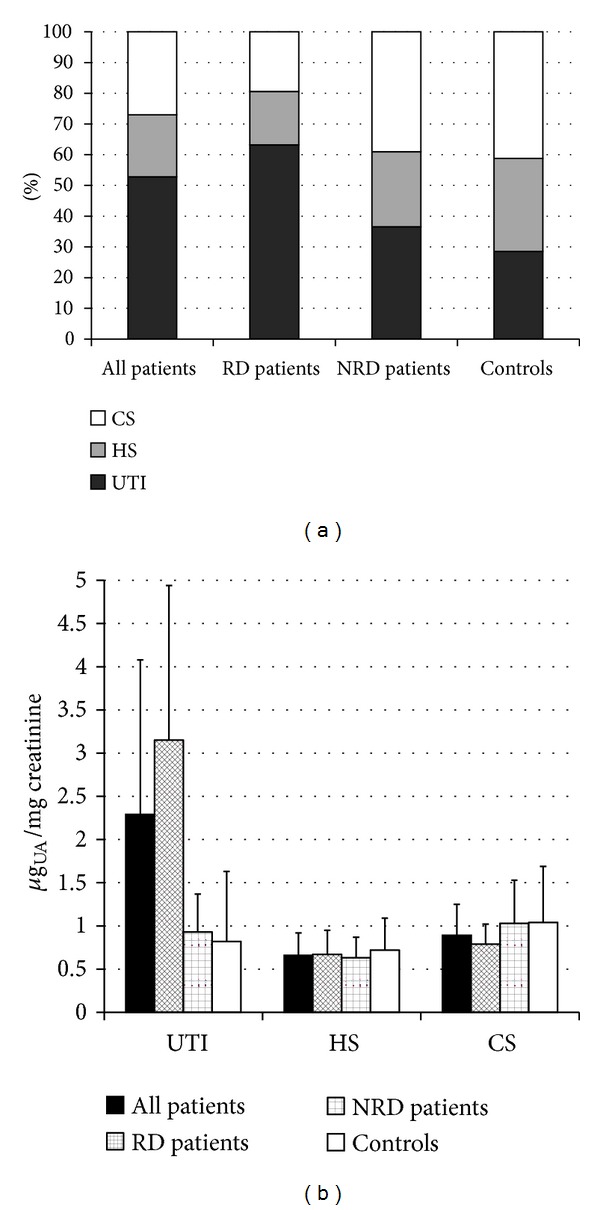
Diagrams reporting percentages (a) and levels (b) of urinary trypsin inhibitor (UTI), heparan sulfate (HS), and chondroitin sulfate (CS) in the totality of patients, patients with renal disease (RD), patients without renal disease (NRD), and controls. UA: uronic acid.

**Figure 3:**
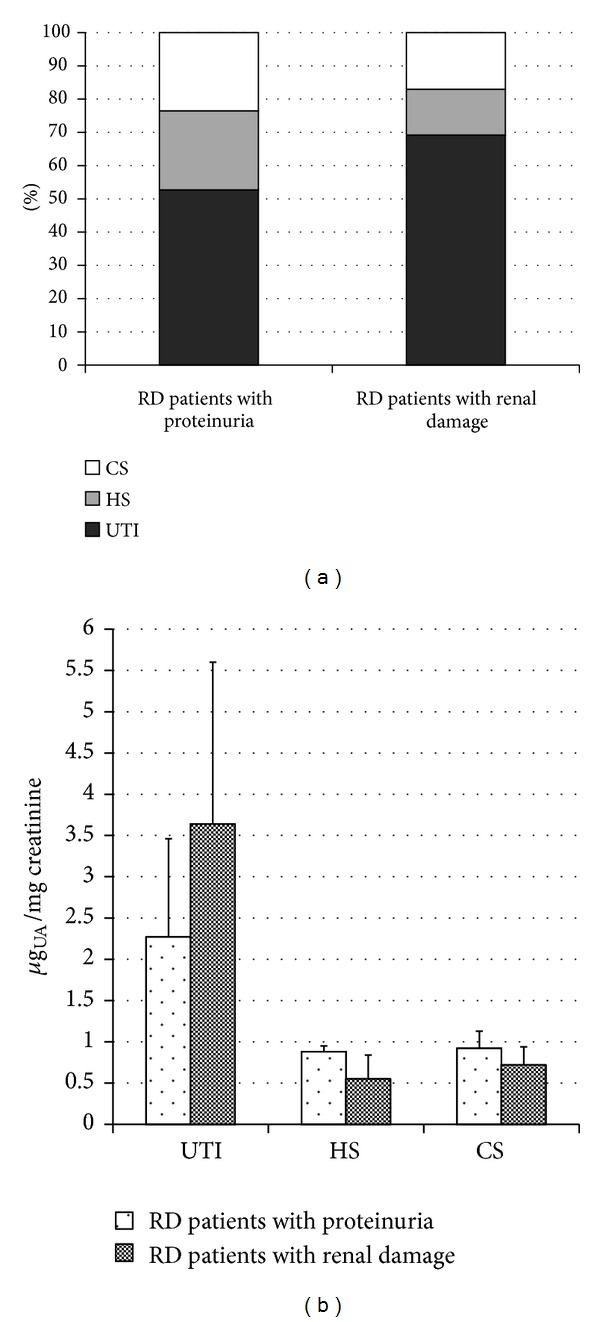
Diagrams reporting percentages (a) and levels (b) of urinary trypsin inhibitor (UTI), heparan sulfate (HS), and chondroitin sulfate (CS) in RD patients with proteinuria and RD patients with renal damage. UA: uronic acid.

**Figure 4:**
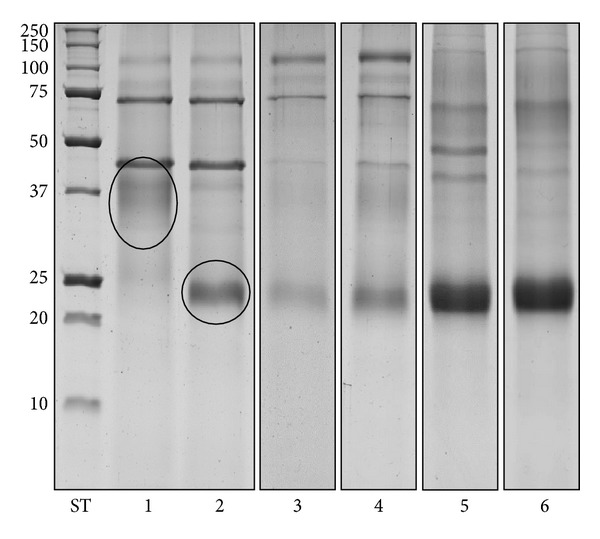
Representative SDS PAGE profiles of nontreated (lane 1) and Chase ABC-treated GAGs-containing fractions from controls (lane 2). Chase ABC-treated GAGs-containing fractions from controls (lane 3), NRD patients (lane 4), RD patients with proteinuria (lane 5), and RD patients with renal damage (lane 6). In lanes 3, 4, 5, and 6, aliquots corresponding to 2 mg of creatinine were loaded. Ovals indicate intact UTI (lane 1) and UTI depleted of chondroitin sulfate chains (lane 2). ST: molecular weight standards (kDa).

Quali-quantitative GAGs analyses were conducted in plasma and urine from 24 Fabry's patients and 43 control subjects. Patients were sorted in RD patients, either with only proteinuria (*n* = 5) or with overt renal damage (*n* = 6), and NRD patients, with no evidence of chronic renal damage (*n* = 13). Qualitative analysis by electrophoresis on cellulose acetate (Figure 1) followed by image analysis allowed evaluating percentages of each purified urinary glycosaminoglycan/proteoglycan, namely, urine bikunin (UTI), HS, and CS. Quali-quantitative results with regard to urine are reported in Table 2 and Figure 2. Hexuronate content analysis evidenced higher urinary GAGs levels (+48%) in patients group in respect to controls (*P* = 0.03). After sorting the group of patients as mentioned previously, it was evident that the major differences found had to be ascribed to the occurrence of renal damage since NRD patients showed urinary GAGs levels not significantly different from controls (*P* = 0.99). The patients group showed quite different electrophoretic profiles with respect to controls (Table 2, Figure 2). After integration, quali-quantitative results evidenced levels of urine bikunin 2.8 times higher in the patients group in respect to controls (*P* = 0.005). Notably, this difference was significant for RD patients only, who showed about 3.8 times higher levels of urine bikunin as compared with control subjects (*P* = 0.0001). To evaluate if urine bikunin levels in RD patients were associated with the degree of renal impairment, the results for RD patients were analyzed after sorting the group in RD patients with only proteinuria (early renal impairment) and RD patients with overt renal damage (Table 3, Figure 3). In this respect, no major differences were evidenced either in GAGs/bikunin levels or in their distribution among the two subgroups, indicating that the increase of bikunin excretion in RD patients is likely an early biochemical event that occurs at the onset of renal impairment. In order to verify if urine bikunin was in its intact form, we performed SDS-PAGE analysis on GAGs/bikunin fractions as a whole and after chondroitin sulfate removal by chondroitin ABC lyase treatment. In this respect, no significant bikunin fragmentation was evidenced in urine samples from either patients or controls (Figure 4). Furthermore, the SDS-PAGE analysis allowed confirming the higher urine bikunin levels in RD patients.

**Figure 5:**
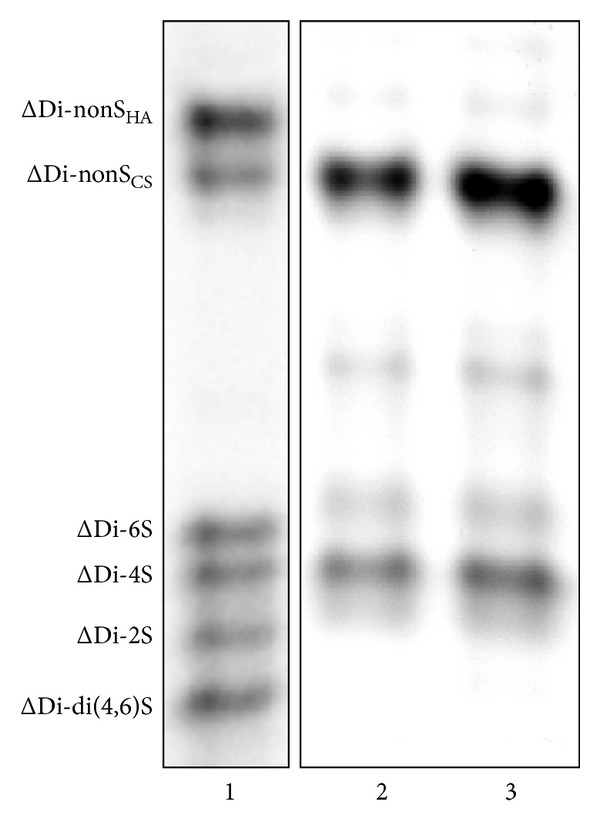
Representative FACE profiles of fluorophore-labeled unsaturated disaccharides (ΔDi) obtained from plasma CS isomers of both controls (lane 2) and Fabry's patients (lane 3). Lane 1: mixture of commercial standard ΔDi (ΔDi-nonS_HA_, 2-acetamido-2-deoxy-3-O-(4-deoxy-*α*-L-threo-hex-4-enepyranosyluronic acid)-4-D-glucose; ΔDi-nonS_CS_, 2-acetamido-2-deoxy-3-0-(4-deoxy-*α*-L-threo-hex-4-enepyranosyluronic acid)-4-D-galactose; ΔDi-6S, 2-acetamido-2-deoxy-3-0-(4-deoxy-*α*-L-threo-hex-4-enepyranosyluronic acid)-6-O-sulpho-D-galactose; ΔDi-4S, 2-acetamido-2-deoxy-3-0-(4-deoxy-*α*-L-threo-hex-4-enepyranosyluronic acid)-4-O-sulpho-D-galactose; ΔDi-mono2S, 2-acetamido-2-deoxy-3-O-(4-deoxy-2-O-sulpho-*α*-L-threo-hex-4-enepyranosyluronic acid)-D-galactose; ΔDi-di(4,6)S, 2-acetamido-2-deoxy-3-O-(4-deoxy-*α*-L-threo-hex-4-enepyranosyluronic acid)-4,6-O-sulpho-D-galactose).

**Figure 6:**
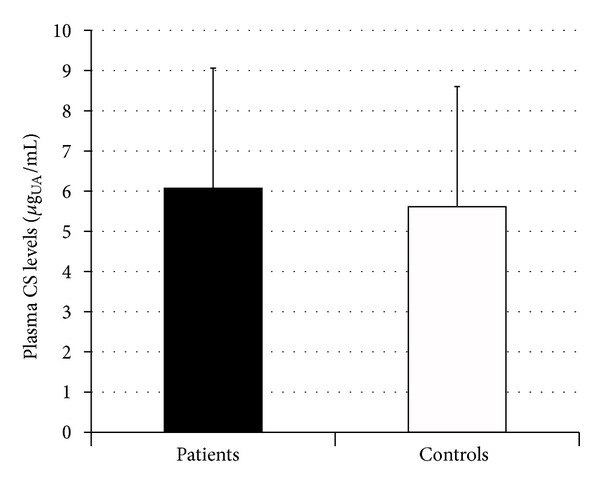
Diagram showing levels of plasma chondroitin sulfate isomers in Fabry's patients and controls. UA: uronic acid.

**Figure 7:**
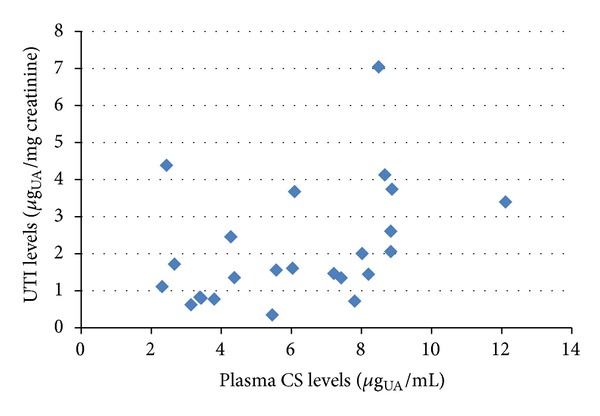
Scatter plot showing levels of urine bikunin (UTI), as *μ*g of uronic acid (UA) per mg of creatinine, in relation to plasma CS isomers levels, as *μ*g of UA per mL of plasma, in Fabry's patients.

To rule out the possibility that higher urine bikunin levels in patients could be ascribed, at least partly, to higher levels of plasma bikunin, we assayed plasma CS isomers by FACE analysis evidencing no differences in either plasma CS levels or charge density between patients and control subjects (Table 4, Figures 5 and 6). Furthermore, no correlation between plasma CS and urine bikunin levels (Figure 7) or between serum creatinine and urine bikunin levels was found suggesting a direct kidney involvement in the higher UTI excretion of Fabry's patients. In absence of overt infection, eleven FD patients out of twenty-four (45.8%) presented with at least one marker of inflammation altered in serum (ESR or CRP, or *α*-1 and *α*-2 globulins). These markers were altered in only 10% of controls. 

## 4. Discussion

Fabry's disease is a multisystemic disorder in which progressive renal impairment, along with cardiac and central nervous system involvement, plays a major role in lowering life quality and expectancy [3, 4]. In a retrospective study on Fabry's patients with renal involvement, Branton et al. showed that 50% of patients had proteinuria by 35 years of age and 100% by 52 years of age [24]. Moreover, 50% of male patients presented with chronic renal insufficiency (CRI) by 42 years of age. These authors evidenced that after the development of CRI, there was a rapid decline in glomerular filtration rate leading to end-stage renal disease within 4.1 years. The enzyme replacement therapy (ERT) seems to represent a valid tool to partly counteract the natural progression of Fabry's disease in combination with renoprotective treatments, such as ACE inhibitors, which are known to be effective in slowing disease progression in other chronic proteinuric kidney diseases [25, 26]. It has also been shown that ERT may be effective in preserving normal renal function in children [27]. Nevertheless, in FD, the diagnosis of an early renal dysfunction is likely of primary importance to aid the clinician in decision making, in designing therapeutic interventions, and in following the natural disease progression or the effects of specific treatments [28].

This paper is the first report to point out that urine bikunin levels are significantly higher in FD patients with renal impairment compared to healthy controls. This finding suggests that the amount of this proteoglycan in urine, as well as proteinuria, could represent an early biomarker of renal impairment in Fabry's patients, useful in monitoring renal functionality also in those patients without overt renal damage.

Interestingly, in our study also several FD patients treated with ERT showed elevated levels of bikunin in urine. Although in this study we did not plan a formal design aimed to evaluate the urinary bikunin levels at baseline and after a period of ERT, this finding may be supportive of several clinical data indicating that ERT may change significantly the natural progression of the disease if started before the establishment of irreversible organ lesion [25, 29].

Plasma and urine levels of bikunin are related to its anti-inflammatory activity [18], and several studies have associated high plasma and/or urine levels of this proteoglycan with various diseases exhibiting chronic inflammation [11–16]. Notably, several clinical and laboratory findings, as the occurrence of episodic and unexplained fever in some patients with FD, and/or the increased serum levels of ESR, CRP, and *α*1- and *α*2-globulin (observed also in 45.8% of our patients with FD) indicate the likely occurrence of both systemic and local inflammation in this pathology [30, 31]. The activation of the inflammatory biochemical pathways in FD, as it may occur in other lysosomal storage disorders (LSDs), is probably related to secondary inappropriate activation of the immune system, in response to storage, resulting in chronic inflammation [32, 33]. It is noteworthy that in LSDs, the occurrence of systemic inflammation contributes to pathogenesis, predates the onset of clinical signs, and may determine the appearance in plasma and/or urine of secondary metabolites which may act as biomarkers that could be useful in following disease progression and may become a target for adjunctive therapy [32, 33]. Importantly, in FD, the ERT is frequently only partially effective in many patients, either due to a late start of this therapy or because of the secondary activation of biochemical mechanisms other than glycosphingolipids storage that also contribute to the pathogenesis of FD. A better understanding of the secondary biochemical pathways involved in FD pathogenesis, as the ones involved in chronic inflammation, may foster the discovery of new therapeutic approaches, adjunctive to ERT, potentially of benefit in this pathology.

Since in our Fabry's patients no correlation was found between plasma CS and urine bikunin levels and no differences were evidenced in plasma CS level/structure between patients and controls, the origin of higher levels of bikunin in urine may imply a direct kidney involvement. Recent RT-PCR analysis studies documenting that several human organs, including the kidney, express bikunin fit this possibility [34]. Moreover, no significant correlation was found between urine bikunin levels and serum creatinine; thus the only impairment of renal function in FD patients seems insufficient to explain higher urine bikunin levels. Nevertheless, the origin of urine bikunin levels and the mechanisms by which urine levels are elevated in our Fabry's patients remains unclear and need to be further evaluated. 

## 5. Conclusions

Our data indicate that urine bikunin levels may be an early biomarker of renal impairment in patients with FD. Moreover, higher urine levels of this secondary metabolite in FD patients suggest the secondary activation, in response to glycosphingolipids storage, of biochemical pathways related to inflammation. Further studies are necessary to test our findings in a larger cohort of FD patients and to investigate any existing correlation between urine bikunin levels, progression of FD, and changes in response to ERT. 

## Figures and Tables

**Table 1:** Clinical and genetic features of Fabry's patients.

Patient	Age	Gender	Renal involvement	GLA mutations	*α*-galactosidase A (nmol/mL/h)*	ERT
1	59	F	RD	Cys172Tyr	1.70	Y
2	26	F	RD	Cys172Tyr	4.68	Y
3	30	M	NRD	Cys172Tyr	3.70	Y
4	38	M	NRD	Cys172Tyr	5.30	Y
5	33	M	proteinuria	Cys172Tyr	2.80	Y
6	27	M	RD	Cys172Tyr	3.40	Y
7	46	F	RD	846_847delTC	3.50	Y
8	24	F	proteinuria	846_847delTC	3.50	Y
9	20	F	proteinuria	846_847delTC	3.30	Y
10	58	F	RD	846_847delTC	3.90	Y
11	36	M	proteinuria	846_847delTC	0.48	Y
12	36	F	NRD	Arg112His	2.80	N
13	61	F	NRD	Gln57Arg	11.10	N
14	62	F	NRD	Gln57Arg	14.80	N
15	23	F	NRD	Gln57Arg	10.40	N
16	58	F	NRD	Gln57Arg	9.80	Y
17	53	F	NRD	Asp313Tyr	15.40	Y
18	52	F	NRD	Arg227Gln	8.10	N
19	31	M	NRD	Arg227Gln	2.50	Y
20	42	M	RD	Arg227Gln	0.10	Y
21	32	M	proteinuria	Arg227Gln	0.70	Y
22	25	M	NRD	Arg227Gln	2.50	Y
23	19	F	NRD	Arg227Gln	6.60	Y
24	43	F	NRD	IVS3+G>A	9.70	Y

NRD: no-renal disease; RD: renal disease; ERT: enzyme replacement therapy; Y: under ERT therapy; N: no ERT therapy.

*At the time of diagnosis.

**Table 2:** Urine GAGs/UTI levels and distribution in Fabry's patients and healthy control subjects.

	All patients (*n* = 24)	RD Patients (*n* = 11)	NRD Patients (*n* = 13)	Controls (*n* = 43)	All patients versus Controls (*P*)	RD Patients versus NRD patients (*P*)	RD Patients versus Controls (*P*)	NRD Patients versus Controls (*P*)
Uronic acid (*μ*g_UA_/mg Cr)	3.83 ± 1.69	4.61 ± 1.63	2.60 ± 0.88	2.59 ± 1.59	**0.03**	**0.009**	**0.004**	0.99

UTI (%)	52.8 ± 20.9	63.2 ± 18.3	36.6 ± 12.9	28.5 ± 17.6	**0.0009**	**0.004**	**<0.0001**	0.29
HS (%)	20.1 ± 9.1	17.4 ± 10.0	24.4 ± 5.6	30.3 ± 9.3	**0.003**	0.113	**0.002**	0.14
CS (%)	27.0 ± 14.4	19.4 ± 9.0	39.0 ± 13.2	41.2 ± 11.9	**0.004**	**0.002**	**<0.0001**	0.70

UTI (*μ*g_UA_/mg Cr)	2.29 ± 1.79	3.15 ± 1.79	0.93 ± 0.44	0.82 ± 0.81	**0.005**	**0.006**	**0.0001**	0.74
HS (*μ*g_UA_/mg Cr)	0.66 ± 0.26	0.67 ± 0.28	0.63 ± 0.24	0.72 ± 0.37	0.57	0.77	0.71	0.58
CS (*μ*g_UA_/mg Cr)	0.89 ± 0.36	0.79 ± 0.23	1.03 ± 0.50	1.04 ± 0.65	0.38	0.18	0.23	0.97

GAGs/PGs levels are reported as *μ*g of uronic acid (UA) per mg of creatinine.

UTI, HS and CS levels are calculated from total UA content and relative percentages of each GAG.

RD: renal disease (proteinuria/renal damage); NRD: no-renal disease.

Significant differences are reported in bold (*P* < 0.05).

**Table 3:** Urinary glycosaminoglycans/UTI levels and distribution in Fabry's patients presenting with either only proteinuria or renal damage.

	RD Patients with proteinuria (*n* = 5)	RD Patients with renal damage (*n* = 6)	(*P*)
Uronic acid (*μ*g_UA_/mg Cr)	4.08 ± 1.40	4.91 ± 1.77	0.44

UTI (%)	52.7 ± 13.6	69.2 ± 18.8	0.16
HS (%)	23.8 ± 8.9	13.8 ± 9.2	0.11
CS (%)	23.5 ± 4.8	17.0 ± 10.3	0.27

UTI (*μ*g_UA_/mg Cr)	2.27 ± 1.19	3.64 ± 1.96	0.24
HS (*μ*g_UA_/mg Cr)	0.88 ± 0.07	0.55 ± 0.29	0.05
CS (*μ*g_UA_/mg Cr)	0.92 ± 0.21	0.72 ± 0.22	0.18

GAGs/PGs levels are reported as *μ*g of uronic acid (UA) per mg of creatinine.

UTI, HS and CS levels are calculated from total UA content and relative percentages of each GAG.

**Table 4:** Plasma CS isomers levels and structure in Fabry's patients and healthy control subjects obtained by FACE analysis of fluorophore-labeled unsaturated disaccharides.

	Patients (*n* = 24)	Controls (*n* = 43)	Patients versus Controls (*P*)
CS isomers (*μ*g_UA_/mL plasma)	6.07 ± 2.96	5.61 ± 2.99	0.66
*CS charge density (%)	41.9 ± 5.9	42.8 ± 8.6	0.72

*CS charge density was evaluated as ratio between ΔDi-4S and the sum of ΔDi-nonS and ΔDi-4S.
